# A cross-cultural study on emotion expression and the learning of social norms

**DOI:** 10.3389/fpsyg.2015.01501

**Published:** 2015-10-02

**Authors:** Shlomo Hareli, Konstantinos Kafetsios, Ursula Hess

**Affiliations:** ^1^The Laboratory for the Study of Social Perception of Emotions, Interdisciplinary Center for Research on Emotions – Department of Business Administration, University of HaifaHaifa, Israel; ^2^Department of Psychology, University of CreteRethymno, Greece; ^3^Department of Psychology, Humboldt-Universität zu BerlinBerlin, Germany

**Keywords:** emotion expressions, social signals, normative behavior

## Abstract

When we do not know how to correctly behave in a new context, the emotions that people familiar with the context show in response to the behaviors of others, can help us understand what to do or not to do. The present study examined cross-cultural differences in how group emotional expressions (anger, sadness, neutral) can be used to deduce a norm violation in four cultures (Germany, Israel, Greece, and the US), which differ in terms of decoding rules for negative emotions. As expected, in all four countries, anger was a stronger norm violation signal than sadness or neutral expressions. However, angry and sad expressions were perceived as more intense and the relevant norm was learned better in Germany and Israel than in Greece and the US. Participants in Greece were relatively better at using sadness as a sign of a likely norm violation. The results demonstrate both cultural universality and cultural differences in the use of group emotion expressions in norm learning. In terms of cultural differences they underscore that the social signal value of emotional expressions may vary with culture as a function of cultural differences, both in emotion perception, and as a function of a differential use of emotions.

## Introduction

Imagine that you watch a group of people. They are taking turns doing a task and when suddenly one person does the task differently, the others look angry. What would you conclude? In a study using such a scenario, participants concluded that if someone wanted to be part of the group, they should do the task like the previous members of the group did it, not like the last member (Hareli et al., 2013). Yet, when the others reacted with sadness, participants were less sure what the proper behavior should be. Thus, the emotions shown by onlookers are one signal people can use to learn how to behave in a new social context.

### Emotion Expressions as Social Signals of Norm Violation

Hareli et al. (2013) focused on anger as a strong signal toward the normativeness of a behavior. The authors grounded their argument on appraisal theory (e.g., Frijda, 1986; Scherer, 1987). Specifically, according to appraisal theories of emotion, emotions are elicited and differentiated through a series of appraisals of (internal or external) stimulus events based on the perceived nature of the event (e.g., Frijda, 1986; Scherer, 1987). Negative emotions such as sadness, anger, and fear are characterized by appraisals of goal obstruction/unpleasantness. That is, these emotions occur when something undesirable happened. For anger, one additional relevant appraisal relates to whether the event is congruent with prevalent norms. As observers can reconstruct appraisals as they apply to a situation (Robinson and Clore, 2002), they can “reverse engineer” or reconstruct the relationship between the person and the event based on the emotion expressed (Frijda, 1986; Weiner, 2006; Hareli and Hess, 2010, 2012). That is, a person who sees an angry other will know that this person encountered an event that was not only undesirable but specifically incongruent with the person’s norms – even if the observer does not know anything else about the emoter and the situation within which the emotion occurred (Hareli and Hess, 2010).

In fact, any event that is appraised as obstructing a person’s goals or as undesirable might be indicative of a problem with the actor’s behavior as well (Scherer, 1987, 1999; Roseman et al., 1990). However, these appraisals simply reflect that something undesirable happened without pointing to norm violations in particular (Scherer, 2001). Thus, these appraisals are a more indirect and less specific sign of non-normative behavior. Consequently, sadness, which signals goal obstruction/unpleasantness but not norm violation, should be less informative regarding norms.

It should be noted that observers are able to deduce a group’s norm just by witnessing uniform behavior by its members (see also, Milgram et al., 1969). Thus, the simple fact that one behavior occurred more often than the other can be indicative of a norm. But even though the uniformity of the behavior as such is a sufficient cue to the norm, it is frequently not used as such (Miller and Prentice, 1996).

The goal of the present study was to assess whether the social signal value of anger generalizes across cultures. Different scenarios are possible, leading to different alternate hypotheses. First, anger is always a potent social signal of social norm violation, sadness a less potent one, and statistical information even less as found by Hareli et al. (2013). That is, no cultural differences will be found. Second, in cultures in which the expression of anger is endorsed to a lesser degree, anger should be a less potent signal of norm violation and this effect should be directly mediated by the perception of anger, yet, sadness should still remain a less potent signal compared to anger. Third, in cultures in which the social meaning of anger is different, anger should be a less potent signal of norm violation with a potential shift in the relative ability of sadness and statistical information to signal norm violations. The rationale for these potential alternative hypotheses is detailed in what follows.

### Cross-cultural Differences in Emotion Perception

The use of bystanders’ emotional reaction to an event to deduce social norms depends essentially on whether these emotional reactions are in fact noticed and decoded. Specifically, if anger serves as a social cue to norm violation, then the perception that a norm violation occurred and the learning of the correct norm should be directly mediated by the degree to which anger is perceived.

Research on cultural differences in the decoding of emotions generally concludes that so-called basic emotions, which include both anger and sadness, are indeed recognized across cultures at above chance levels (Elfenbein and Ambady, 2002; Hess and Thibault, 2009). Yet, these findings refer to highly prototypical intense facial expressions shown without context and even for these facial emotion expressions, differences in decoding accuracy across countries have been observed (Elfenbein and Ambady, 2002). As everyday emotions are typically more subtle and non-prototypical (Motley and Camden, 1988; Ekman, 2003) and occur within a context (Hess and Hareli, 2014) differences in decoding accuracy are very likely. For the decoding of such more subtle expressions, decoders take recourse to stereotype knowledge and socio-cultural norms regarding the “proper” display of emotion expressions when trying to understand these expressions (Kirouac and Hess, 1999).

In fact, there are strong cultural differences in emotional display rules (Matsumoto et al., 2008), that is, the social rules that guide the appropriate display of emotion expressions (Ekman and Friesen, 1971). These differences can in part be related to differences in cultural values such as individualism and collectivism (Matsumoto et al., 2008) but also openness to change (Koopmann-Holm and Matsumoto, 2011) or masculinity (Sarid, 2015) among others. In fact, even though cultural values underpin the establishment of display rules within a culture, it is unlikely that they depend crucially on a single dimension but rather one would expect them to be embedded into a richer cultural fabric. Importantly in this context, social display rules have a converse side in social decoding rules (Buck, 1984; Hess, 2001), such that perceivers tend to be less good at decoding expressions that are proscribed in a given culture. Thus, cultures that differ in anger display rules can also be expected to differ in anger perception.

The present study replicates the study by Hareli et al. (2013) in Germany, Greece, the US, and Israel. These cultures were chosen because they differ with regard to the cultural endorsement of anger. Different underlying social values and motives seem to explain differences in anger display rules between the US and Greece on the one hand and Germany and Israel on the other. In comparison to other individualistic cultures, European Americans in the US tend to avoid negative affect (Koopmann-Holm and Tsai, 2014), which may explain their lower endorsement of anger expressions in comparison to Germans in particular (Koopmann-Holm and Matsumoto, 2011). Therefore, the motivation to shy away from negative affect can be the result of more individualistic concerns to distance from others. On the other hand, Greek participants with higher interdependence tend to show lower attention to negative emotions, including anger expressions, due to collectivism concerns and the keeping of harmony rules (Kafetsios and Hess, 2013).

There are no studies that compare all four countries, but a number of studies exist that allow us to triangulate the likely differences across all four. In a recent study, Hess et al. (in press) found that Greek participants rated spontaneous facial expressions of anger less accurately and less intensely than did German participants. Germans also endorsed anger (as well as sadness) expressions more than US Americans, a finding that the authors relate to differences in openness to change (Koopmann-Holm and Matsumoto, 2011). In turn, the expression of anger is endorsed to a larger degree in Israel than in the US, a difference that has been explained by differences in power distance (Margalit and Mauger, 1984; Grandey et al., 2010). Based on these data, we predicted that anger expressions would be rated more intensely in Germany and Israel, followed by the US and Greece.

With regard to sadness ratings, unlike the US, Germany, and Israel, Greece is a more interdependent country in which the expression of anger is endorsed less and sadness is valued relatively more and also recognized better than in Germany (Hess et al., in press). Also, Greece is higher in uncertainty avoidance, which has been linked to better sadness decoding (Schimmack, 1996). Thus, sadness should be rated more intensely in Greece than in the other three countries and more intensely in Germany than the US.

Consequently, we hypothesized anger to be a strong social signal of norm violation in Germany and Israel but less so in the US and least in Greece. Specifically, as there is evidence that anger is differentially endorsed and perceived in the four different cultures (H1) we expected that, in line with the second alternative above, in cultures in which the expression of anger is endorsed to a lesser degree, anger should be a less potent signal of norm violation and this effect should be directly mediated by the perception of anger (H2). By contrast, sadness should be a still less potent signal compared to anger. However, as there is some evidence that negative emotions in a relational context have different meanings for German and Greek participants (Kafetsios et al., under review), it may be that in this culture sadness will be a relatively more potent signal of norm violation (H3).

Further, we predicted that the potency of cultural display rules as decoding rules depends on the perspective of the observer. Specifically, it may make a difference whether a situation is supposed to be evaluated from a certain social distance as it relates to other people, or if it is to be evaluated from a first person point of view making it directly relevant to the observer (Ham and van den Bos, 2008). Thus, we expected stronger effects when the situation is to be evaluated from a first person point of view making it directly relevant to the observer (H4).

### Overview

Following the design of Hareli et al. (2013), participants were presented with a series of slides that depicted a group of people. In all slides one of the group members was shown drinking tea while the others looked on. The first two slides each showed a different group member holding the teacup in a specific way and the onlookers showed a neutral expression. In the third slide the teacup was held differently and the onlookers either reacted with anger, sadness, or neutrality. The expressions were carefully created to be of medium intensity only. Participants were then asked to: (a) describe the norm in their own words, (b) rate how likely they thought it to be that a norm violation had occurred and (c) rate the emotions shown by the onlookers in the last slide.

As mentioned above, statistical information on the relative frequency of the two behaviors alone can be indicative of the presence of a norm. Yet, as Miller and Prentice (1996) put it, “norm-congruent behaviors are both unremarkable and unlikely to be remarked on” (p. 808). Hence the cup was either held first with one hand in the way commonly done in all four cultures, or with two hands, which should be more salient, as this represents a cultural (but not group) norm violation. We therefore expected statistical information to be more informative when the group norm was to hold the teacup with both hands. Hareli et al. (2013) did not find a significant difference in norm learning as a function of hand position, but the data did show a difference in means congruent with such a possibility. Finally, we varied the personal relevance of the norm by asking participants to adopt either a first or a third person perspective when being asked about the norm. A first person perspective should make the question more personally relevant (Ham and van den Bos, 2008), which should increase motivation and attention. This resulted in a 4 (country) × 3 (last picture emotion expression) × 2 (normative hand position) × 2 (first vs. third person perspective) between subjects design.

## Materials and Methods

### Participants

A total of 149 (84 men, 56%) individuals with a mean age of 32 years (*SD* = 8) participated in a laboratory setting at the University of Haifa (Israel). Further, 273 (120 men, 44%) individuals with a mean age of 23 years (*SD* = 7) were recruited for an online study using a database of current and former students at the University of Crete (Greece); 261 (84 men, 32%) individuals with a mean age of 26 years (*SD* = 5) were recruited for an online study via the Facebook page of the department of psychology at Humboldt-University, Berlin (Germany), and 452 (247 men, 56%) with a mean age of 33 years (*SD* = 11) were recruited via Amazon Mturk in the US and completed the study.

### Procedure

In Haifa, participants came to the laboratory in groups of up to five. They were greeted, informed consent was obtained and they then completed the same computer task as was used in the online studies. For the online studies, participants received the same information and consented by clicking a button^1^.

The first screen explained that the study was about social perception and that participants would see three photos that documented part of an event. The next slide described the event. Participants were told that recently four members of a group that belonged to a social order, which is concerned with charitable work, had a meeting. The organization was further described as having an old tradition that includes different ceremonies. Participants had to pretend that they were invited to participate in a traditional tea drinking ceremony by that group. During the ceremony, one after the other, each member has to drink tea from his or her cup. Participants were then told that they would see three photos showing the actions of three group members and the other members’ reaction to these actions. They were further told that the photos are presented in the order in which the actions occurred. Participants were warned that the photos would appear for a brief time only and that they would be asked to describe afterward how either someone else who is the next to participate would behave or how they themselves would behave if they were next to participate. The three photos were then presented for 8 s each.

### Stimulus Material

**FIGURE 1:**
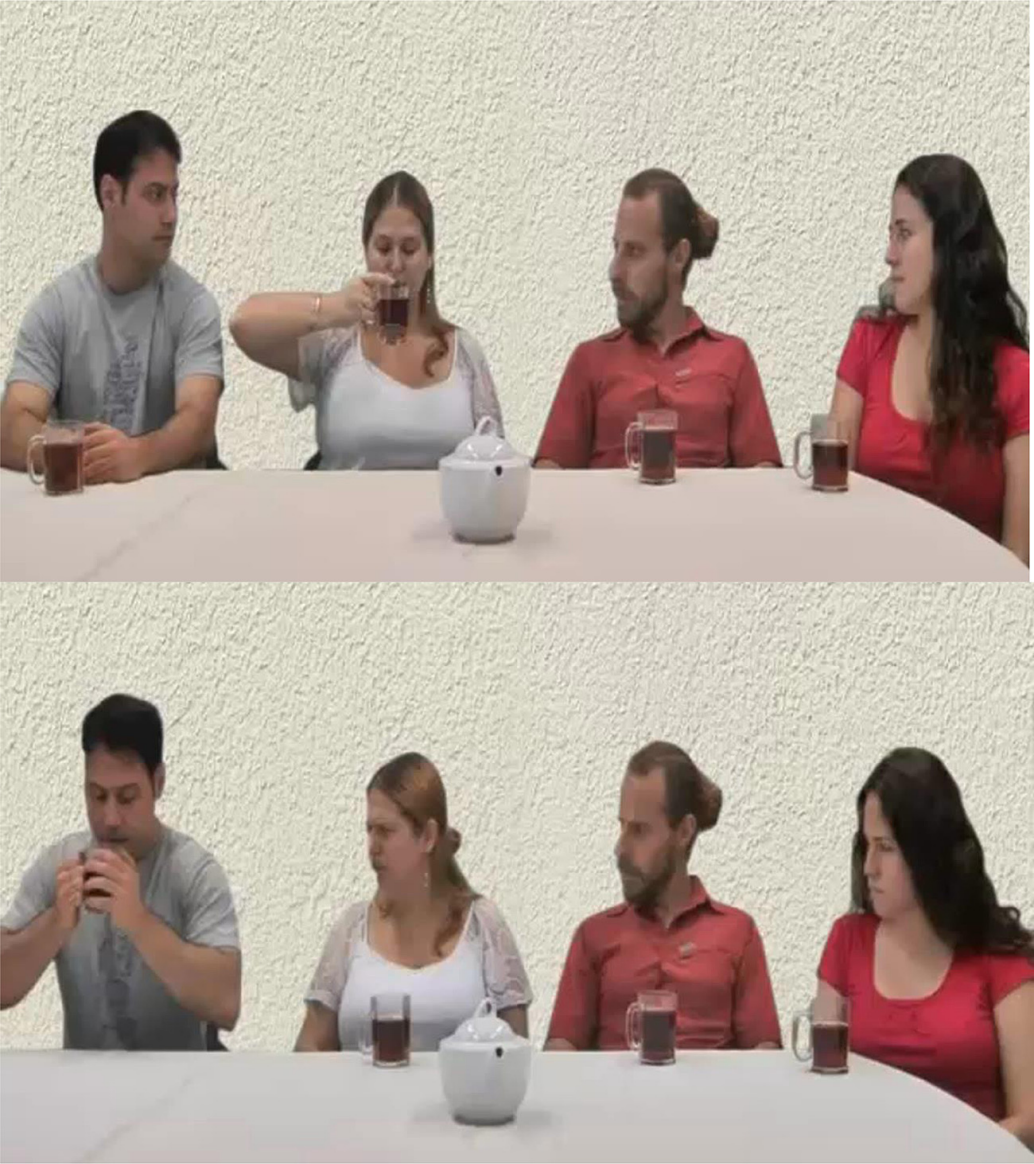
**Example stimulus**.

The stimulus slides were taken from Hareli et al. (2013) and adapted by removing Hebrew writing visible in the slides. The slides showed one of three group members drinking the tea and the others watching and reacting to this behavior. The first two slides each showed a different group member holding the teacup close to the mouth with both hands and the arms raised away from the body. The third group member was shown as holding the teacup only with the right hand. Non drinking group members were always shown looking at the acting person while expressing emotional neutrality when the member held the teacup with two hands (for an example of the stimulus material, see **Figure 1**). In a second condition, the norm was to drink the tea one handed and the norm violation was two handed drinking. Depending on the experimental condition, group members expressed anger, sadness, or emotional neutrality to the non-normative behavior of the last group member.

### Dependent Measures

Following the last photo, participants were requested to answer an open question asking them to report either (a) how the participants would expect an acquaintance who wants to behave according to the “group spirit” would act (the original question asked by Hareli et al., 2013) or (b) how they themselves would act if they wanted to behave according to the “group spirit.”

Participants’ responses to the open question were classified into two categories by two raters. Rare inconsistencies were resolved by discussion. One category included answers that reflected a clear understanding of the norm, such as, “S/he will drink the tea holding the cup with two hands.” The other category included answers that reflected that the participants did not understand the norm, such as, “S/he will sit and look and even drink tea.”

Once they had completed their answer, participants were referred to the last photo and asked to rate to what extent the group members had expressed sadness or anger or seemed indifferent. Finally, participants were asked to rate in two separate questions the extent to which group members saw the behavior of the person holding the cup as violating conventions and to what extent they saw it as violating social laws or norms. As these two questions correlated substantially (*r*_Greece_ = 0.75, *r*_Germany_ = 0.88, *r*_Isarel_ = 0.89, and *r*_USA_ = 0.90) they were combined into one variable named norm violation. These ratings were made on seven-point scales anchored at the extremes, ranging from (0) “not at all” to (6) “very much.”

## Results and Discussion

Because the samples differed with regard to mean age and gender composition, these variables were initially included as covariates in the analyses below. Gender was never significant and age only for ratings of anger (and global intensity which includes anger), such that older individuals rated the expressions as less angry (angry expressions: *r* = -0.14, *p* = 0.007; sad expressions: *r* = -0.17, *p* = 0.001, neutral expressions: *r* = -0.11, *p* = 0.031). None of the ANOVA results changed when the covariates were included and for the mediation analyses the inclusion of the covariates strengthened the effect of anger.

### Emotion Perception

#### Overall Intensity

As we predicted that participants from the four cultures should vary in their ratings of anger (H1) we first assessed whether there were overall differences in the intensity ratings of the emotions expressed. Such differences could be due to culture-specific response styles and hence not specifically related to anger. For this, we summed the emotion ratings across all three scales and conducted a one-way ANOVA with culture as a factor. A significant effect of culture emerged, *F*(3,1141) = 2.87, *p* = 0.036, $\eta_{p}^{2}$ = 0.01, which, however, explained only about one percent of the variance. The overall perceived intensity was highest for Israel (*M* = 6.95, *SD* = 2.52), followed by Germany (*M* = 6.84, *SD* = 2.92), the US (*M* = 6.53, *SD* = 3.24), and finally Greece (*M* = 6.19, *SD* = 3.15). A *post hoc* test revealed that Greece differed from Israel and Germany, which did not differ from each other. Including age as a covariate, a slightly stronger country effect emerged, *F*(3,1125) = 3.82, *p* = 0.010, $\eta_{p}^{2}$ = 0.01, and the ratings for the US and Greece differed as well. In all, any differences in perceived anger intensity between Germany, Israel, and the US were not due to an overall trend to rate expressions less intensely. However, this could be the case for Greece, even though the overall difference between Greece and the other countries is rather small.

### Is Anger Perceived Differently as a Function of Country?

**FIGURE 2:**
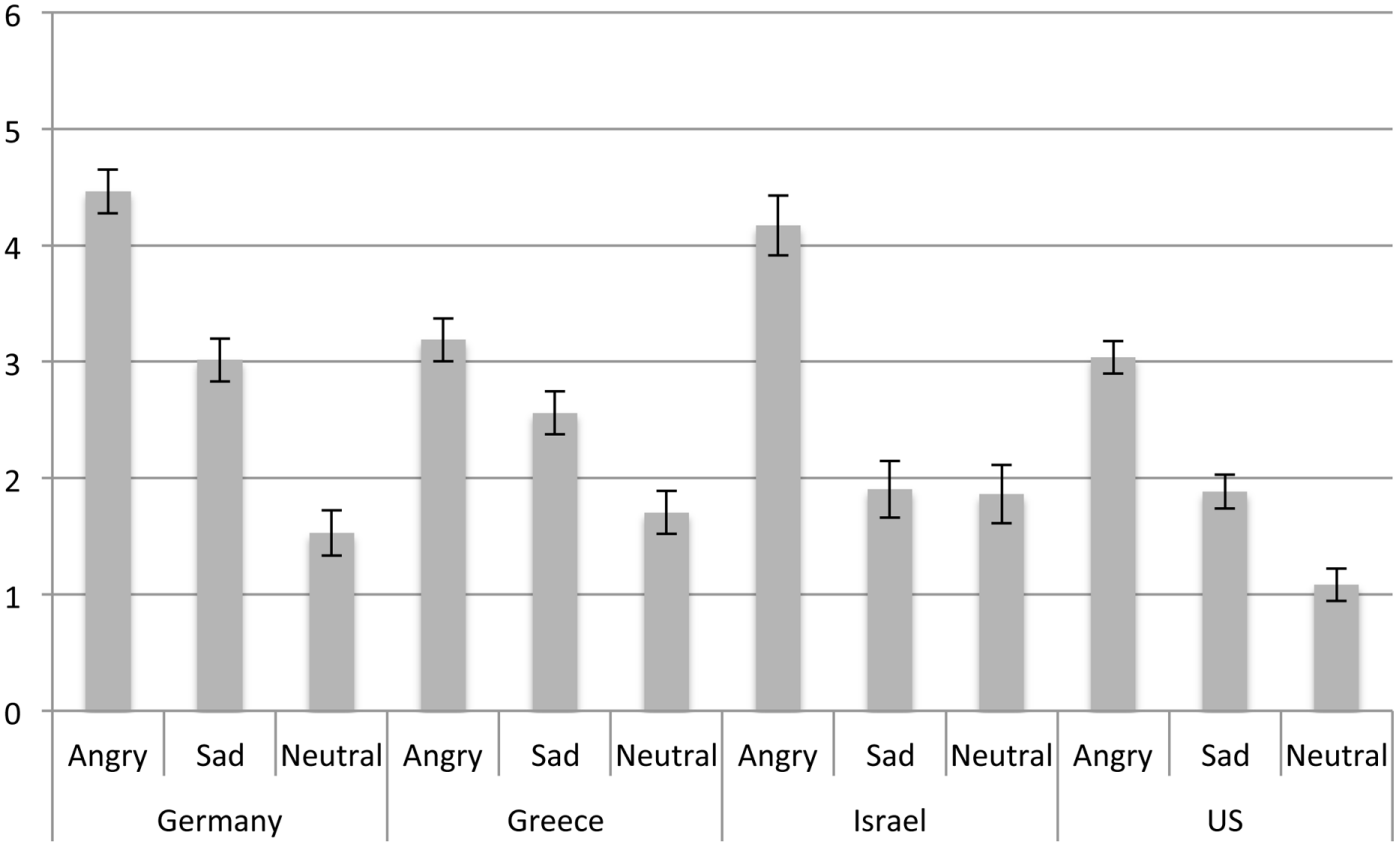
**Anger ratings as a function of emotion expression and country**.

To assess our prediction that participants from the four cultures should vary in their ratings of anger (H1) we conducted an analysis of variance with a 4 (country) × 3 (emotion expressions in the last picture: Anger, Sad, Neutral) between-subjects design on the anger ratings with age as a covariate, *F*(2,1119) = 6.82, *p* = 0.009, $\eta_{p}^{2}$ = 0.01 (for means and standard errors see **Figure 2**). In line with the notion of the universality of emotion expression perception, a significant main effect of last picture expression, *F*(2,1119) = 122.81, *p* < 0.001, $\eta_{p}^{2}$ = 0.18, emerged such that across countries, anger expressions were rated as significantly more angry than sadness expressions, which were rated as significantly more angry than neutral expressions. The significant main effect of country, *F*(3,1119) = 14.96, *p* < 0.001, $\eta_{p}^{2}$ = 0.04, was qualified by a country × last picture expression interaction, *F*(6,1119) = 4.77, *p* < 0.001, $\eta_{p}^{2}$ = 0.03. Specifically, as predicted, *post hoc* tests revealed that anger expressions were rated as more intensely angry in Germany and Israel, which did not differ, than in Greece and the US, which also did not differ (H1).

*Post hoc* comparisons showed that expressions of sadness were rated as most intensely angry in Germany, followed by Greece, which differed only marginally (*p* = 0.066) from Germany and Israel (*p* = 0.071). Sadness expressions were rated as significantly less angry in both Israel and the US, which did not differ. Neutral expressions were rated as least angry in the US compared to Germany, Greece and Israel, which did not differ. The ratings in Germany did not differ from any other country.

In sum, as expected, anger was rated differently as a function of culture (H1) and whether anger was the focal emotion expression. Interestingly, German participants tended to perceive more anger in all three types of expressions. By contrast, participants from the US perceived generally less anger in all three expressions. Greek participants perceived less anger in angry expressions but relatively more anger in the non-angry expressions, suggesting that they perceive emotions as more mixed.

### Are There Differences in the Perception of Sadness and Neutrality?

#### Sadness Intensity

**FIGURE 3:**
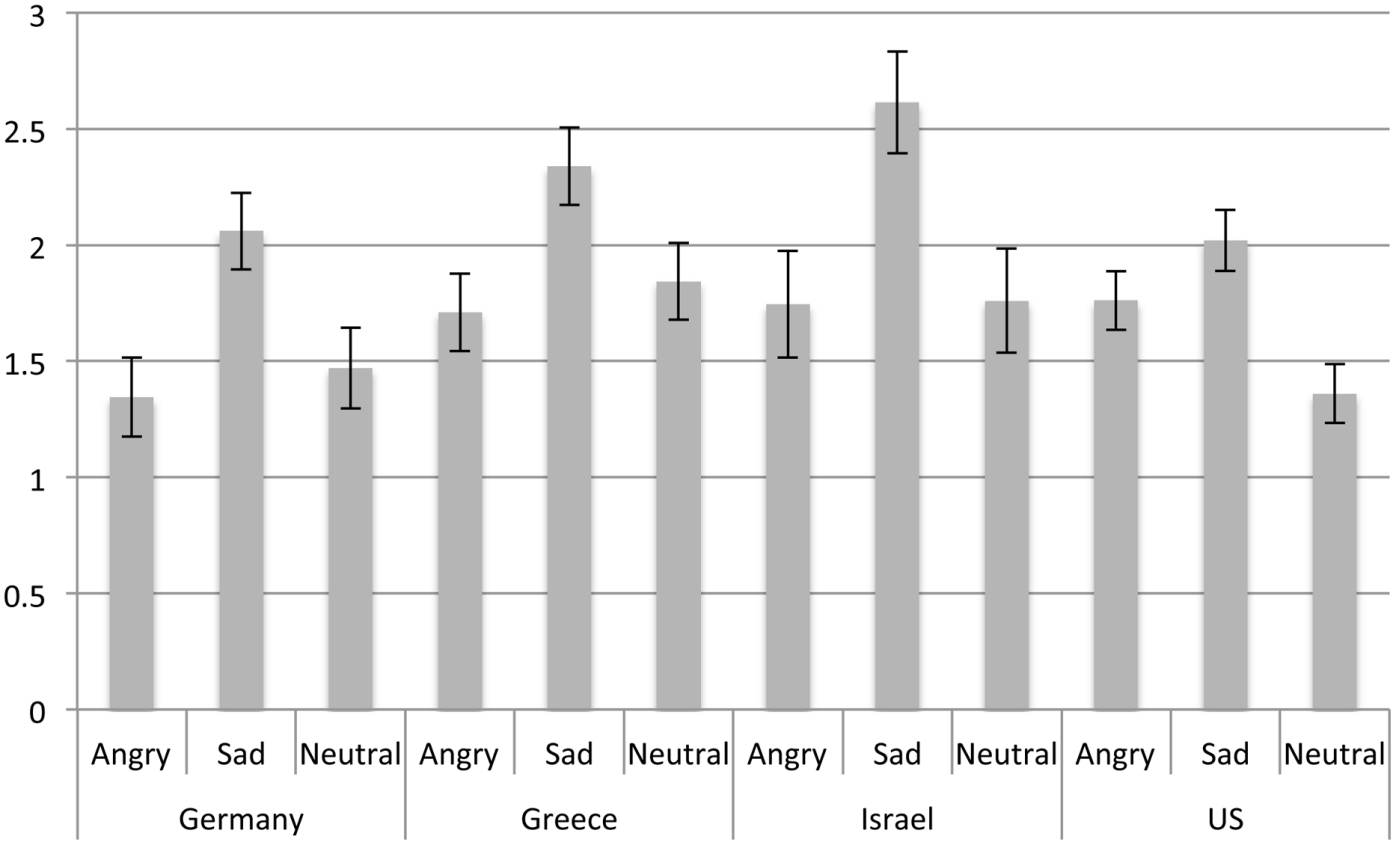
**Sadness ratings as a function of emotion expression and country**.

To assess whether the four countries also differed with regard to their perception of sadness, a 4 (country) × 3 (emotion expressions in the last picture: Anger, Sad, Neutral) analysis of variance was conducted (for means and standard errors see **Figure 3**). Significant main effects of country, *F*(3,1133) = 3.63, *p* = 0.013, $\eta_{p}^{2}$ = 0.01, and last picture emotion, *F*(2,1133) = 17.63, *p* < 0.001, $\eta_{p}^{2}$ = 0.03, emerged such that, again in line with the notion of the universality of emotion perception, sadness expressions were rated as more sad than anger expressions and neutral expressions, which did not differ. Across expressions *post hoc* comparisons showed that Israeli and Greek participants, who did not differ, perceived more sadness than German and US participants, who also did not differ. The finding for Greek participants is congruent with the notion that individuals from countries high in uncertainty avoidance are more accurate in the perception of sadness (Schimmack, 1996), as Greece (100) is highest on this norm. Even though Israel (81) is lower in uncertainty avoidance than Greece it is still considerably higher than Germany (65) and the US (46, numbers refer to Hofstede, 2015), and this may explain the finding for Israel.

#### Indifference Ratings

**FIGURE 4:**
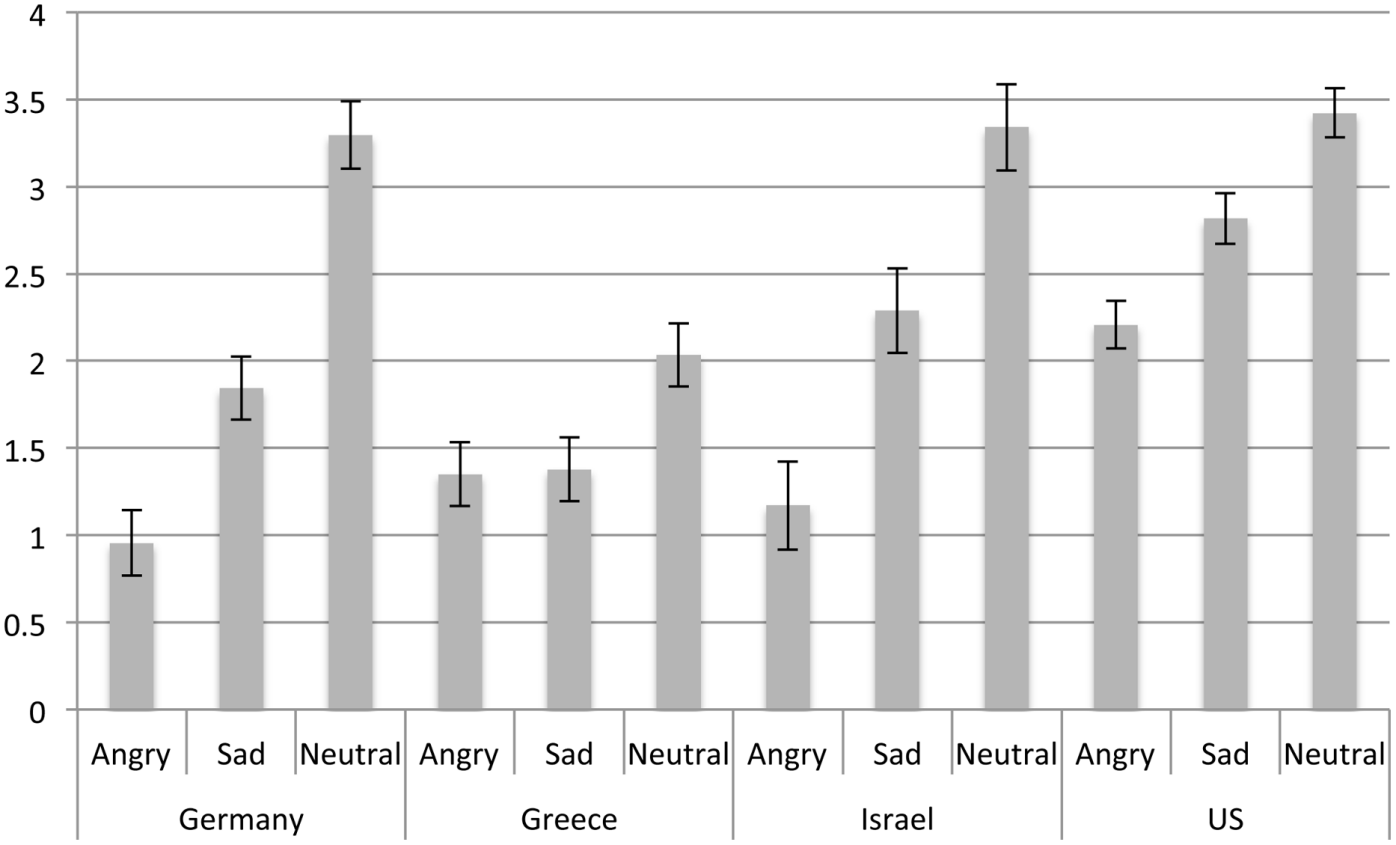
**Indifference ratings as a function of emotion expression and country**.

To assess whether the four countries also differed with regard to their perception of indifference, a 4 (country) × 3 (emotion expressions in the last picture: Anger, Sad, Neutral) analysis of variance was conducted (for means and standard errors see **Figure 4**). Significant main effects of last picture emotion emerged, *F*(2,1133) = 68.87, *p* < 0.001, $\eta_{p}^{2}$ = 0.11, such that neutral expressions were rated as expressing most indifference, followed by sadness and anger expressions, which were rated as least indifferent. The main effect of country, *F*(3,1133) = 27.70, *p* < 0.001, $\eta_{p}^{2}$ = 0.07, was qualified by a last picture emotion × country interaction, *F*(6,1133) = 4.64, *p* < 0.001, $\eta_{p}^{2}$ = 0.02, such that angry expressions were perceived as most indifferent in the US compared to Greece, Israel, and Germany, which did not differ. *Post hoc* comparisons showed that sadness expressions were also rated as most indifferent by participants from the US as well as least indifferent by Greek participants, with Israeli (*M* = 2.29, *SE* = 0.25) and German participants at intermediate levels. Finally, neutral expressions were rated as least indifferent by Greek participants compared to German, Israeli, and US participants, which did not differ.

Thus overall, participants from the US rated all expressions as indicating relatively high levels of indifference, which matches their lower ratings of anger and sadness across expressions. By contrast, Greek participants rated expressions generally as showing less indifference, however, they tended to see relatively higher levels of sadness throughout. German participants rated in particular anger expressions as low in indifference, which matches their higher ratings of anger. Overall, Israeli participants tended to be most accurate in their perception.

### Are There Differences in Norm Learning?

An open question assessed whether participants had spontaneously learned the tea drinking norm. Importantly, they were only asked how they or another person would act without any verbal hint toward a possible norm transgression. The 0 (inaccurate) -1 (accurate) codes were analyzed using a 4 (country) × 3 (emotion expressions in the last picture: Anger, Sad, Neutral) × 2 (normative hand position: first vs. second hands) × 2 (perspective: first vs. third person) between-subjects design.

**FIGURE 5:**
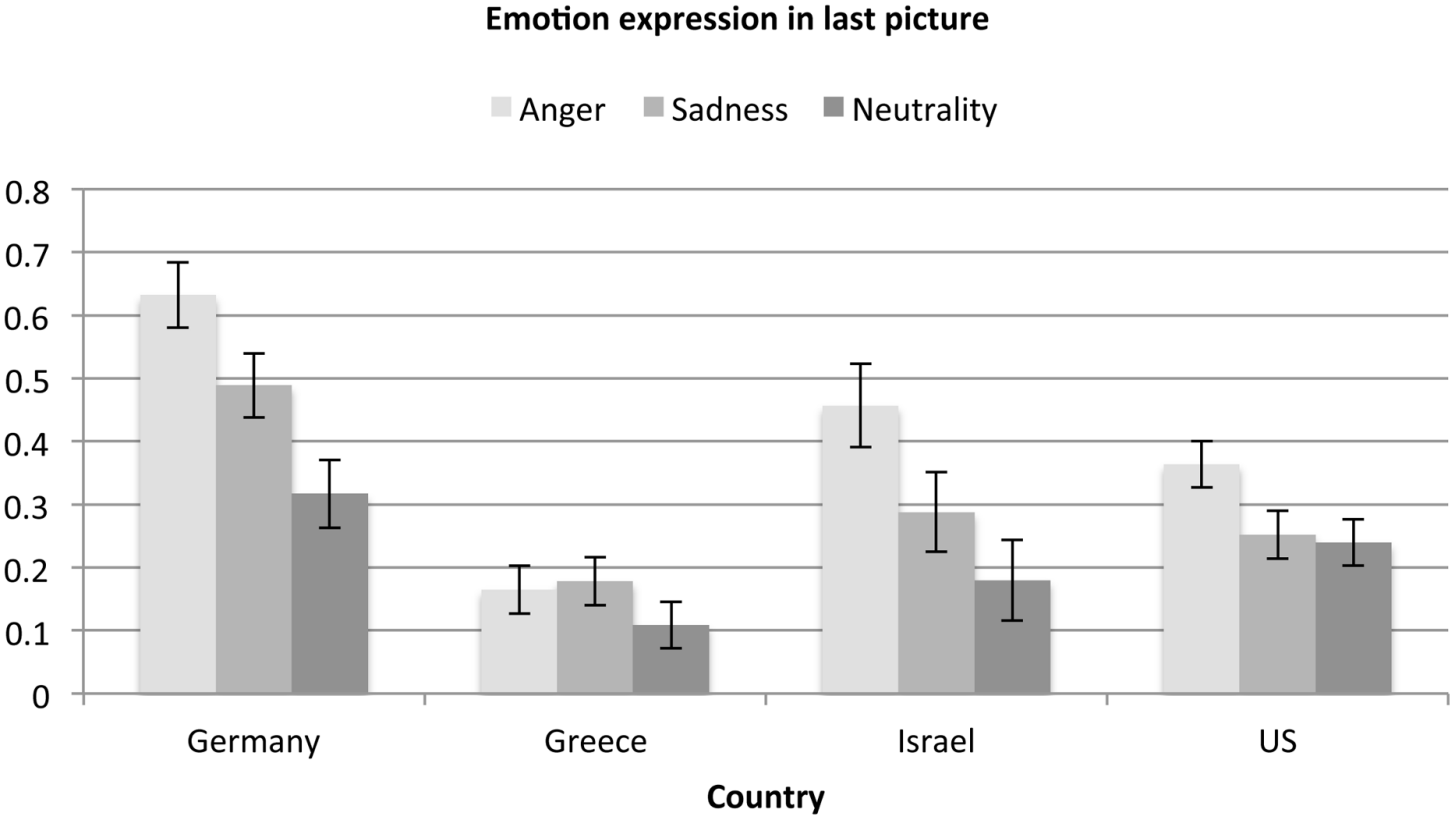
**Norm learning as a function of country and emotion expression in the last picture**.

Significant main effects of country, *F*(3,1078) = 26.22, *p* < 0.001, $\eta_{p}^{2}$ = 0.07, emotion, *F*(2,1078) = 15.29, *p* < 0.001, $\eta_{p}^{2}$ = 0.03, and normative hand position, *F*(1,1078) = 46.15, *p* < 0.001, $\eta_{p}^{2}$ = 0.04, were qualified by emotion by normative hand position, *F*(2,1078) = 6.32, *p* = 0.002, $\eta_{p}^{2}$ = 0.01, and emotion by country, *F*(6,1078) = 2.44, *p* = 0.024, $\eta_{p}^{2}$ = 0.01, interactions, respectively. Overall, *post hoc* comparisons showed that participants from Germany were most accurate (*M* = 0.48, *SE* = 0.03), and participants from Greece were least accurate (*M* = 0.15, *SE* = 0.04), with Israel (*M* = 0.30, *SE* = 0.04) and the US (*M* = 0.29, *SE* = 0.02), which did not differ, at intermediate levels (for means and standard errors see **Figure 5**).

### Are There Differences in the Appraisal of Norm Violation?

We further assessed whether participants – even if they may not be accurate in reporting the actual norm – did nonetheless realize that a norm violation occurred. For this, we conducted a 4 (country) × 3 (emotion expressions in the last picture: Anger, Sad, Neutral) × 2 (normative hand position: first vs. second hands) × 2 (perspective: first vs. third person) analysis of variance on the appraisal of norm violation.

**FIGURE 6:**
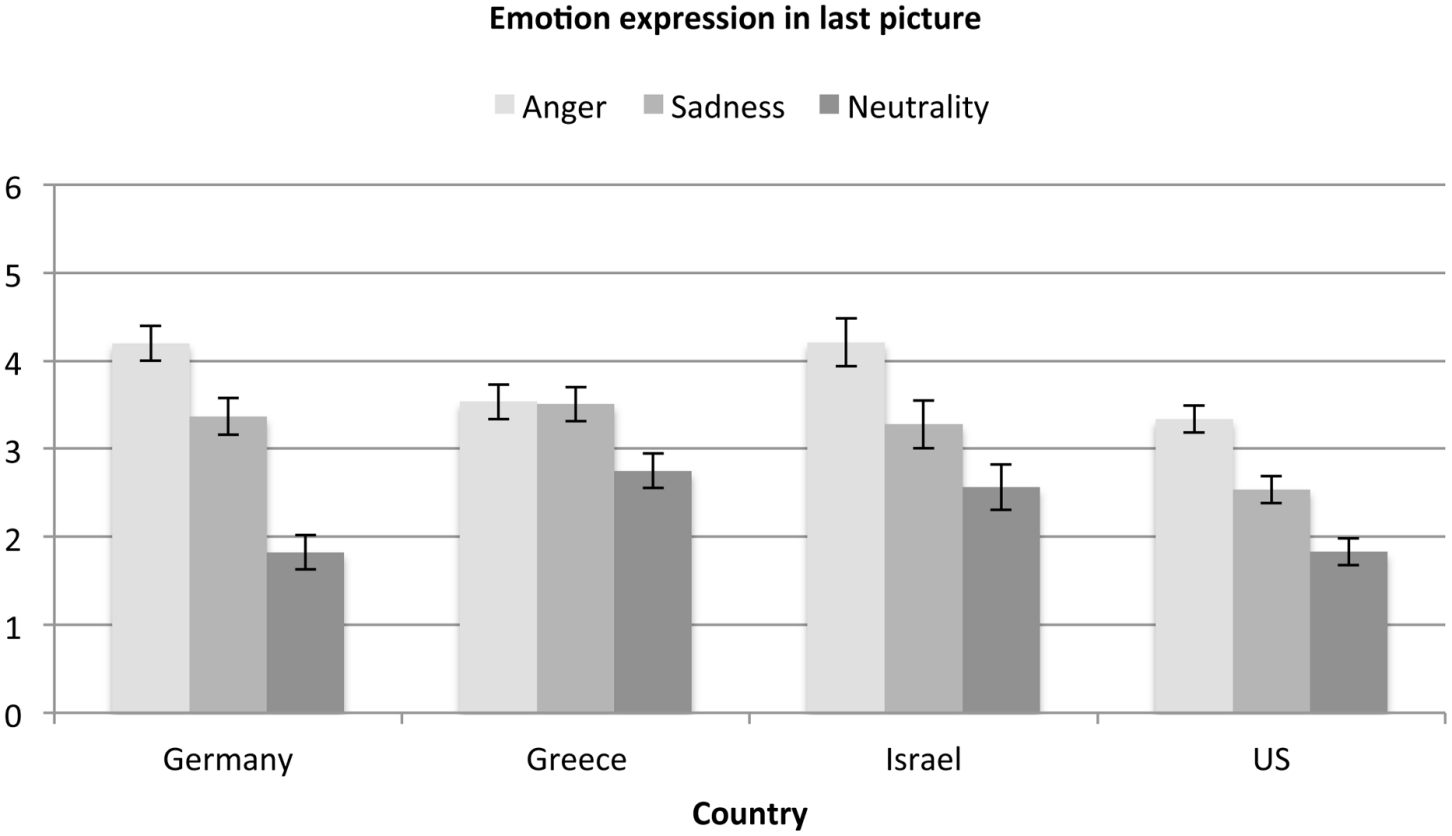
**Appraisal of norm violation as a function of country and emotion expression in the last picture**.

A significant main effect of emotion expression, *F*(3,1097) = 56.98, *p* < 0.001, $\eta_{p}^{2}$ = 0.09, emerged such that, overall and as expected, participants considered a norm violation to be most likely when the group had shown anger (*M* = 3.82, *SE* = 0.11), followed by sadness (*M* = 3.17, *SE* = 0.11), and finally neutrality (*M* = 2.24, *SE* = 0.10). This effect was qualified by an emotion expression by country interaction, *F*(6,1097) = 3.19, *p* = 0.004, $\eta_{p}^{2}$ = 0.02, such that this pattern emerged significantly for Germany, Israel and the US, whereas for Greece ratings for sadness and anger did not differ (see **Figure 6**).

Across countries the likelihood that a norm violation occurred also differed. A significant main effect of country emerged, *F*(3,1097) = 11.70, *p* < 0.001, $\eta_{p}^{2}$ = 0.03, such that this likelihood was considered lowest in the US (*M* = 2.57, *SE* = 0.09), compared to Germany (*M* = 3.13, *SE* = 0.12), Greece (*M* = 3.26, *SE* = 0.11 and Israel (*M* = 3.35, *SE* = 0.15), which did not differ in the *post hoc* comparisons. Thus, even though Greek participants were especially inaccurate in learning the norm, they were still aware of a norm violation. Notably though, for Greek participants both sadness and anger were equally good signals of norm violation. This finding and the finding that norm learning did not differ between anger and sadness conditions in the Greek sample support Hypothesis 3. This may point to the possibility that negative emotions have a different meaning in relational contexts in an interdependent country as suggested by Kafetsios et al. (under review). Greek participants were also, together with Israel, better at concluding that a norm violation had occurred based on statistical information only.

### Are Differences in Norm Learning and Norm Violation Appraisal Mediated by Emotion Perception?

Hareli et al. (2013) found that participants were better at deducing the norm when the group showed anger in response to the norm violation, followed by sadness while least accuracy was predicted for neutral expressions. In the present study, this pattern emerged again for Israel and for Germany but only for Germany were all three conditions significantly different from each other. For Israel, anger led to a better rate of deducing the norm than did sadness or neutrality. Sadness, however, was not better than neutrality (see **Figure 5**). For Greece there was no difference in accuracy between anger and sadness, whereas for the US there was no difference between sadness and neutrality. This pattern largely matches the pattern of anger perception reported above. We therefore assessed whether accuracy was mediated through anger perception as predicted by Hypothesis 2.

To assess whether anger perception mediated both norm learning accuracy and the appraisal of norm violation (H2), we regressed these variables on the emotion ratings separately for each emotion expression condition as well as across emotion expression conditions with age as a covariate (see **Table 1**). Age was only significant for norm learning, such that older individuals learned the norms more readily when the individuals in the last picture showed either sadness or anger. When age was included as a covariate the beta for the effect of anger ratings on norm learning improved slightly. There was no effect of age on norm appraisal.

**Table 1:** Significance levels and βs as a function for last picture emotion expression as a function of expression condition.

	β						
	*F*	*p*	*r*^2^	Anger	Sadness	Neutrality	Age
**Overall**				
Norm learning accuracy	41.97	0.001	0.13	0.35^∗∗∗^	-0.04 (ns)	-0.06^∗^	0.09^∗∗∗^
Appraisal of norm violation	295.21	0.001	0.51	0.56^∗∗∗^	0.17^∗∗∗^	-0.17^∗∗∗^	-0.04^t^
**Anger in last picture**				
Norm learning accuracy	20.10	0.001	0.18	0.36^∗∗∗^	0.01 (ns)	-0.13^∗^	0.11^∗^
Appraisal of norm violation	85.45	0.001	0.48	0.59^∗∗∗^	0.11^∗∗^	-0.16^∗∗∗^	-0.03 (ns)
**Sadness in last picture**				
Norm learning accuracy	16.95	0.001	0.16	0.35^∗∗∗^	0.04 (ns)	-0.15^∗∗^	0.17^∗∗∗^
Appraisal of norm violation	95.57	0.001	0.51	0.52^∗∗∗^	0.18^∗∗∗^	-0.23^∗∗∗^	-0.05 (ns)
**Neutrality in last picture**				
Norm learning accuracy	3.11	0.016	0.03	0.19^∗^	0.02 (ns)	0.08 (ns)	0.03 (ns)
Appraisal of norm violation	65.65	0.001	0.42	0.39^∗∗∗^	0.29^∗∗∗^	-0.07 (ns)	-0.05(ns)

*^∗^p < 0.05, ^∗∗^p < 0.01, ^∗∗∗^p < 0.001.*

The same pattern of significant effects emerged for all conditions^2^. All models were significant for both norm learning accuracy and the appraisal of norm violation. Norm learning accuracy was significantly positively predicted by anger rating intensity and negatively by ratings of indifference but not by sadness ratings. The appraisal of norm violation was also positively predicted by anger intensity ratings and negatively by indifference ratings but also positively by sadness ratings.

Together, these findings suggest that anger is a strong social signal of norm violation even for expressions that do not include anger as the focal emotion. Ratings of indifference are indicative of a perception of a lack of emotionality of the group. According to appraisal theories of emotion (e.g., Scherer, 1987), emotions are only elicited by events that are relevant to the emoter. Hence, when the group seemed indifferent, participants were more likely to conclude that nothing noteworthy had happened, which explains why these ratings are negatively related to perceptions of norm violation and norm learning accuracy. Interestingly, sadness intensity ratings only significantly predicted the appraisal of norm violation but not norm learning accuracy. This is supportive of the notion that an appraisal of goal obstruction/unpleasantness as indexed by sadness is a sign that something is wrong, but is less indicative of what exactly is wrong.

### Does Taking a First Person Perspective Increase Norm Learning and Appraisals of Norm Violation?

A significant main effect of perspective, *F*(1,1078) = 6.43, *p* = 0.011, $\eta_{p}^{2}$ = 0.01, emerged for norm learning, such that across conditions and countries, participants’ descriptions were more accurate when the first person perspective was adopted (*M* = 0.34, *SE* = 0.02), then when the third person perspective was used (*M* = 0.27, *SE* = 0.02), confirming the notion that personal relevance increases norm learning. As for norm learning, a significant main effect of perspective emerged for norm violation appraisals as well, *F*(1,1097) = 6.90, *p* = 0.009, $\eta_{p}^{2}$ = 0.01. Specifically, participants considered it more likely that a norm violation had occurred when they adopted a first person perspective (*M* = 3.34, *SE* = 0.08 vs. *M* = 2.92, *SE* = 0.09) suggesting that personal relevance also increases the awareness of a norm violation. Together these findings suggest that participants paid more attention when the task was made personally relevant to them (H4).

### Does the Salience of the Hand Position Impact on Norm Learning and Appraisals of Norm Violation?

**FIGURE 7:**
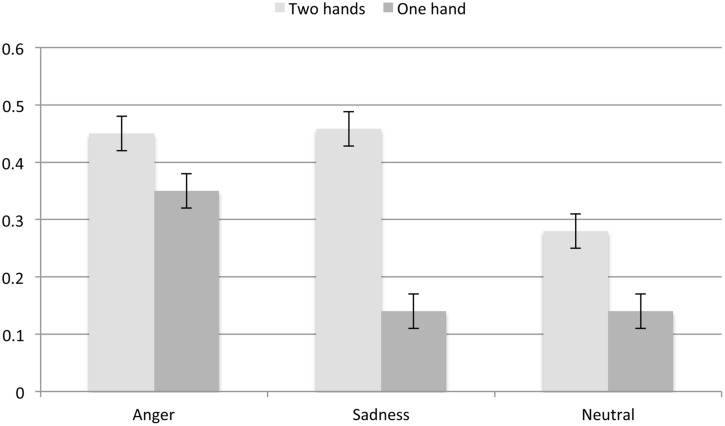
**Norm learning as a function of hand position and emotion expression in the last picture**.

We had predicted that the norm violation would be more salient when the norm describes a hand position that varies from the culturally normative hand position. This, because culturally normative behavior is generally unremarkable (Miller and Prentice, 1996). Given that the situation has a certain level of complexity, it is more likely that the culturally “deviant” hand position would be salient to the observer and hence the switch to the other hand position would be more noticeable. As such, participants should be better at using simple statistical information when the normative behavior deviates from the cultural norm of drinking tea from a cup held one-handedly rather than two-handedly. In fact, participants were overall better at learning the norm when the norm was to hold the cup with both hands (*M* = 0.40, *SE* = 0.20) rather than one hand (*M* = 0.21, *SE* = 0.02). As expected, no significant difference as a function of normative hand position emerged for the anger expression condition, *t*(373) = 1.77, *p* = 0.078, *d* = 0.18. By contrast, for both the sadness, *t*(375) = 6.84, *p* < 0.001, *d* = 0.71, and neutral conditions, *t*(372) = 3.66, *p* < 0.001, *d* = 0.38, participants were better when the norm involved both hands (see **Figure 7**), thus, for the two conditions in which participants were overall less accurate, the cultural normativeness of the hand position made a difference, however, this difference was larger for sadness than for neutrality. For appraisals of norm violation, by contrast, the main effect of normative hand position was not significant, *F*(1,1097) = 1.57, *p* = 0.211, $\eta_{p}^{2}$ = 0.00, suggesting that the salience of the hand position did not tip participants off as to whether a norm violation had occurred. In sum, the culturally normative hand position was most effective as a cue when participants were trying to understand the exact norm and onlookers did not show anger.

## General Discussion

The present study was conducted to assess the role of emotion expressions as social signals of norm violation in a cross-cultural context. Because anger expressions are based on an appraisal of norm violation, we had predicted that anger is a powerful signal of norm violation (H2). Yet, cross-cultural research suggests that the perception of anger varies with cultural norms and decoding rules (e.g., Grandey et al., 2010; Koopmann-Holm and Matsumoto, 2011; Hess and Hareli, 2014). More recent research also suggests that emotions may vary in their social-relational meaning between independent and interdependent cultures (Kafetsios et al., under review). These considerations allow for the possibility that the social signal value of anger expressions varies with culture. This can be either as a function of cultural differences in emotion perception, based on display/decoding rules, or as a function of a differential use of emotions in different cultures. The present research provides evidence for both notions.

First, even though across the four cultures clear evidence for the universality of emotion perception emerged, in that anger expressions were rated as most angry, sadness expressions as most sad, and neutral expression as most indifferent, there were nonetheless substantial between-culture differences in emotion ratings (H1). In particular, German participants were especially prone to perceive anger, whereas Greek participants were more likely to perceive sadness, replicating observations by Hess et al. (in press). Also, participants from the US were more likely to perceive the expressers as indifferent. These findings suggest that members of different cultures are differentially sensitive to specific emotions. At the same time, Israeli participants overall differentiated best between the three types of expressions, which may reflect an in-group advantage (Elfenbein et al., 2007) as the expressions were created in Israel.

Importantly, and as expected, these cultural differences in emotion perception predicted cultural differences in norm learning accuracy and appraisals of norm violation (H2). Specifically, across all emotion expression conditions, ratings of anger were positive and ratings of indifference were negative predictors of both norm learning accuracy and appraisals of norm violation. That is, independent of whether the expressions were angry, sad, or neutral, participants were more likely to learn the norm and perceive the norm violation, to the degree that they considered the expression as showing anger. As such, anger was found to be a potent signal of norm violation not only when shown as a focal emotion, but also to the degree that it was detected within other emotion expressions. Consequently, in cultures in which social norms are more lenient with regard to the expression of anger (Germany and Israel) participants were more likely to describe a norm accurately and appraise the situation as likely to involve a norm violation, when the group reacted with anger to the norm violation.

Appraisals of norm violation, but not norm learning, were also predicted by ratings of sadness, however, the effect was notably weaker. This suggests that an expression linked to an appraisal of goal obstruction/unpleasantness, which signals that something undesirable has happened can, in the right context, be a signal of norm violation as well, yet a less powerful one. Notably, Greek participants, who perceived anger to a lesser degree and sadness to a higher degree than members of the other cultures, were better at using sadness as a sign of a likely norm violation, but not for norm learning. As such, they were aware that a norm was violated but not why. US participants by contrast seemed to be better able to use statistical information for norm learning.

Two additional factors had been varied, normative hand position and the perspective that the participants were asked to assume. Even though the effect of hand position did not reach statistical significance in the study by Hareli et al. (2013), the pattern of means was suggestive of such an effect. In fact, common sense suggests that a behavior that violates a cultural norm (such as the polite way of holding a teacup) is more salient than a behavior that conforms to the norm, which in fact may be invisible to the casual observer (Miller and Prentice, 1996). Thus, it may be expected that group norm effects interact with cultural norms, such that a group norm that conflicts with the cultural expectations for proper behavior is more readily apparent and learned more easily. This was indeed the case but notably only for behaviors that were reacted to with sadness or neutrality. That is, when a behavior was reacted to with anger, participants were not advantaged by the additional salience of the behavior. This suggests that seeing anger is a sufficiently clear signal that observers are able to recreate the scene in their mind to a degree that allows them to describe the group norm even when it is not salient. Interestingly, the salience of the behavior was most effective for expressions reacted to with sadness. As sadness signals that something is wrong, salience may be what is needed to figure out what it is that is wrong. This role of salience is also supported by the fact that emotion expression and hand position combine to affect norm learning but not the appraisal of norm violation, which can be made based on the emotion expression information alone.

Finally, we also varied whether participants adopted a first or a third person perspective when describing the norm. For both norm learning and the appraisal of norm violation, a main effect of perspective emerged. As no interaction effects were found, this seems to simply suggest that participants paid more attention when the task was made more personally relevant by adopting a first person perspective. This suggests that apart from the factors noted above, motivation is also a significant factor in norm understanding (H4).

This factor also seems to play a role in understanding why German participants were especially good, across conditions, at norm learning. This difference cannot be explained by emotion rating tendencies alone as these were not very different between Israel and Germany. However, it has been suggested and demonstrated that members of different cultures are in fact differentially sensitive to norms. Gelfand et al. (2011) distinguishes between “tight” cultures, which have strong norms and a low tolerance for deviant behavior and “loose” cultures, which have weak norms and a high tolerance for deviant behavior. The expectation is that members of tighter cultures should be more concerned about behaving according to norms and more concerned about social sanctioning because of the lower tolerance by tight cultures for deviance (Gelfand et al., 2006). As Germany is higher in cultural tightness than the other three countries in our study (Gelfand et al., 2011), it is thus likely that German participants were especially sensitive to the norm violation as well as more motivated to learn the correct norm.

The study had some limitations. Notably, the samples varied somewhat in age and gender composition. However, when gender was used as a covariate it was not significant. Even though gender differences in emotion recognition accuracy are frequently reported, they are not always found. In fact, research in this domain has also pointed to strong motivational effects as underlying the observed gender differences in recognition accuracy (Ickes et al., 2000). As only one image had to be rated, it may well be that gender was less influential. By contrast, age was found to influence emotion perception for anger such that older participants tended to rate all images as less angry. The effect was rather weak, but is noteworthy as only 1% of the participants was over 60 and hence the age of our participants was within a range where differences in emotion perception accuracy have not been previously reported. However, the task here regards the sensitivity to anger, that is, the intensity with which anger was perceived and not the question of whether anger was mislabelled, which is the focus of most emotion recognition studies. Nonetheless, when age was included as a covariate, the effect of anger on norm learning was slightly higher and no effect on norm appraisal was found.

Participants in the present study were asked to assume to want to join the group. It is interesting to speculate on the effect of anger expressions by group members in reaction to a norm violation on that motivation. Thus, Heerdink et al. (2013) found that group members whose opinions deviated from a group felt rejected when the group reacted with anger. This finding suggests that even though anger expressions are helpful in facilitating people’s integration into a group by allowing them to learn the relevant group norms, they may do so at a cost. This is a question for future research.

In sum, the present study showed that across cultures anger is a potent signal for norm violation. However, whether this signal is used to its full extent depends on the observers’ culturally determined sensitivity to anger expressions and the culturally determined use of such expressions. In this sense, the present research demonstrates both cultural universality and cultural differences. Thus, in the terms of Norenzayan and Heine (2005) the use of anger as a signal of norm violation is a functional universal, in that anger perception is a cognitive tool found in all cultures that serves the same function in all cultures, but is used to different degrees in different cultures. In this vein, the present research also underlines the importance of considering both universality and cultural variation when studying emotions. The strong impact of social norms and values on the perception of emotion is congruent with appraisal theory of emotion, which sees emotions determined by such rules and norms. It is therefore only logical that their perception is also influenced by such rules and norms. Focussing exclusively on universality ignores this basic fact but focusing exclusively on cultural variation ignores the existence of basic cognitive tools that all humans dispose of – but simply may use to different extends.

## Conflict of Interest Statement

The authors declare that the research was conducted in the absence of any commercial or financial relationships that could be construed as a potential conflict of interest.

## Footnotes

1 In previous research we found no differences between emotion ratings effectuated in a laboratory setting and those using the web-based version for the same ratings once appropriate checks on diligence were done. For the MTurk sample, a question regarding the understanding of the instructions and a control item (if you read this, please move the slider to the left) were included to detect automated answers. For all studies, questionnaires that did not include an answer to the open question were considered as incomplete and discarded.

2 In one exception, indifference ratings did not significantly predict the appraisal of norm violation in the neutral emotion expression condition.
